# Specific learning disabilities and associated emotional-motivational profiles: a study in Italian university students

**DOI:** 10.3389/fpsyg.2024.1365980

**Published:** 2024-07-26

**Authors:** Marika Iaia, Francesca Vizzi, Maria Diletta Carlino, Marco Turi, Chiara Valeria Marinelli, Paola Angelelli

**Affiliations:** ^1^Lab of Applied Psychology and Intervention, Department of Experimental Medicine, University of Salento, Lecce, Italy; ^2^Department of Human and Social Sciences, University of Salento, Lecce, Italy; ^3^Learning Science hub, Department of Humanities, University of Foggia, Foggia, Italy

**Keywords:** specific learning disabilities, emotional profile, motivational profile, university students, Italian sample

## Abstract

In this study, we analyzed the emotional and motivational aspects characterizing the profile of university students with Specific Learning Disorders (SLD). We assessed 61 university students, 32 with SLD (age = 23.6) and 29 in the control group (age = 23.00). The results highlighted that individuals with SLD exhibit higher levels of anxiety and depression and lower resilience compared to the control group. The Multidimensional Perfectionism Scale – Short Form, which explores perfectionism, did not reveal differences between the groups. Conversely, lower scores emerged in SLD students for the intrinsic motivation sub-scales of the Academic Motivation Scale. This indicates less engagement in studying out of personal cognitive curiosity. The Self-Regulated Knowledge Scale – University, which measures various cognitive strategies, showed significantly lower scores in the SLD group for knowledge linking, knowledge training, and knowledge critique. This suggests a lower frequency with which SLD students attempt to connect new knowledge with what they already possess, apply their knowledge, ask questions, and critically analyze what they have learned. Therefore, psychological and motivational consequences are evident in this population and can impact well-being and quality of life.

## 1 Introduction

Specific Learning Disorders (SLD) constitute a class of neurodevelopmental disorders characterized by enduring challenges in the domain of learning, specifically in areas such as reading, writing, and arithmetic, as defined by the DSM-5 (American Psychiatric Association, 2013). These difficulties often tend to co-occur, but can also occur in isolation. They are related to probable common genetic bases and partially shared anomalies in the neurofunctional circuits involved in these abilities. SLD are disorders confined to specific cognitive domains and do not affect broader cognitive functioning (AA.VV, 2022). However, their consequences can still be pervasive, impacting many aspects of cognitive functioning, as well as personal and social adaptation. In developmental age and in a language with transparent orthography such as Italian, reading and writing abilities show continuous development regarding both accuracy and speed. Dyslexic children increase their reading speed by an average of one-third of a syllable per second per year (Tressoldi et al., 2001). In addition to making more spelling errors, they tend to pause more frequently during text composition compared to their typically developing peers (Sumner et al., 2013). Their orthographic lexicon is limited, with difficulty in spelling stimuli that require the use of the lexical procedure and a later optimization of lexical spelling compared to sublexical one (Angelelli et al., 2010, 2016; Notarnicola et al., 2012; Marinelli et al., 2021). Regarding mathematical learning difficulties, there may be disturbances in various areas involved in the acquisition of numerical and calculation skills: knowledge of calculation procedures, knowledge of numbers, the ability to recall numerical facts, and multiplication tables (Lucangeli and Tressoldi, 2001). These data imply that the gap between typical readers and dyslexic individuals increases over the years and that the comorbid condition could lead to a more severe clinical picture than the manifestation of the isolated disorder. Therefore, SLD, especially if not promptly recognized, can lead to a cascade of secondary problems in the psychopathological sphere (forms of anxiety/depression, etc.), or behavioral and social issues (self-regulation, social interaction, etc.) (Livingston et al., 2018). Considering that disorder, whether in milder and concealed forms or through compensatory mechanisms, can persist throughout one’s entire life and remain a source of stress, its impacts on psychological well-being can manifest at any age, exhibiting significant differences both in terms of quantity and quality (Dahle et al., 2011). Some studies have shown that children and adolescents with dyslexia, in addition to facing problems with failing grades and school dropout, are at a higher risk of developing social problems (Wiener and Schneider, 2002; Lipka et al., 2006) and emotional disturbances (Capozzi et al., 2007; Goldston et al., 2007). Anxiety is the most commonly reported emotional symptom in children with dyslexia: recent studies (Nelson and Harwood, 2011) highlight the presence of symptoms related to school-related anxiety in about 70% of individuals with learning difficulties. Among the various motivational aspects involved in the learning processes and the associated experiences, the literature has highlighted crucial concepts such as competence, self-efficacy, attributive style, and self-esteem. In children and adolescents with SLD, there is a more negative self-concept (Tabassam and Grainger, 2002), lower self-esteem not confined to the academic sphere alone but generalized to almost all domains (Polychroni et al., 2006; Marinelli et al., 2016), and greater insecurity about their intelligence, accompanied by a heightened sense of inadequacy (Humphrey and Mullins, 2002; McNulty, 2003). They tend to feel less responsible for their own learning (Anderson-Inman et al., 1996) and they are more likely to persist less or give up tasks at the first sign of difficulty (Bouffard and Couture, 2003). Furthermore, adolescents with SLD report being victims of bullying to a greater extent than their peers with typical development when they do not perceive the presence of meaningful relationships with their classmates, suggesting how a supportive school environment would promote their well-being and learning processes (Marini et al., 2023).

The university experience can sometimes be a significant source of psychological distress (Besser and Zeigler-Hill, 2014; Touloumakos et al., 2016), with a high prevalence of mental disorders in the university student population (Papadatou-Pastou et al., 2015). Despite the occurrence of compensatory processes during development that significantly enhance the performance of individuals with SLD, the underlying biological factors persist and can significantly influence the academic activities of those who choose to pursue higher education (Carroll and Iles, 2006; Klassen et al., 2011). Throughout each school year, the reading speed of students with dyslexia increases by approximately half compared to typical readers, with a greater slowdown after the third year of upper secondary school (Tressoldi et al., 2001). In young adults, there is indeed an improvement in decoding accuracy, but difficulties in speed and automation persist (Martino et al., 2011). Compared to the control readers, dyslexic adults achieve poorer results in measures of word and pseudoword reading speed, phonological awareness, and orthographic knowledge, than in the corresponding accuracy measures (Reis et al., 2020). These results suggest that speed measures are more suitable than accuracy in identifying reading and writing difficulties not only in developmental ages but also in adults, especially those from more transparent orthographies (Re et al., 2011; Suárez-Coalla and Cuetos, 2015; Iaia et al., 2021).

The condition SLD in adults is therefore complex and requires additional personal commitment and appropriate strategies to overcome difficulties, leading to a substantial implication of challenges regarding psychological well-being in general. Therefore, it seems important to promote academic success by strengthening foundational skills and fostering the development of self-esteem, a sense of self-efficacy, and a healthy motivational style and anxiety management. However, despite the relevance of the issue and its significant implications for National Education and Health Systems, according to one analysis conducted during the Istituto Superiore di Sanità (2011) promoted by the Higher Institute of Health and the new Guidelines on the management of Specific Learning Disorders (2022), the studies on the effectiveness of rehabilitative interventions for reducing the severity of the disorder and associated emotional-motivational deficits are insufficient, and further data are needed.

The number of university students with SLD is constantly increasing. National statistical data on the percentage of university students with SLD in Italian public universities have revealed that the incidence for the academic year 2019/2020 is 1.42% (Borgonovi et al., 2022). The rate of academic success holds particular significance in situations that can complicate a student’s university path, such as in the case of SLD (Bonifacci et al., 2016; Matteucci and Soncini, 2021). This evidence underscores the importance of understanding how the performance of these students is influenced by various emotional and motivational factors that play a fundamental role in supporting or hindering their university path (Carmona-Halty et al., 2021).

In the literature, previous studies have shown that university students with dyslexia exhibit lower self-esteem and experience more psychopathological issues. This is particularly evident in terms of anxiety and depression compared to their typically developing peers (Undheim, 2003; Carroll and Iles, 2006; Ghisi et al., 2016). Nelson and Gregg (2012), in fact, highlight that college undergraduates with developmental dyslexia exhibit a higher frequency of symptoms associated with anxiety and depression compared to those transitioning from high school. Depressive symptoms can, for example, manifest as loss of interest, lack of energy, low mood, difficulty concentrating, pessimism, sadness, self-criticism, and alterations in sleep or appetite (Re et al., 2014). Murdaca et al. (2014) underline the essential role of emotional factors, often overlooked in the context of university education, in predicting students’ academic performance. Anxiety and depression related to academic studies are likely contributing factors to the increased dropout rate among university students with learning difficulties (Davis et al., 2009). A recent study (Bonuomo et al., 2023) showed that university students with SLD exhibit low perceived academic competence, academic anxiety, and emotional and interpersonal difficulties. Biasi et al. (2017) have highlighted that the cognitive strategies adopted, the type of motivation, and the perception of self-efficacy influence the learning abilities of university students. Among the various factors, more or less consistent, in predicting the academic performance and persistence of university students, one can identify the motivation for success, academic self-efficacy, and academic skills in terms of cognitive, behavioral, and emotional tools necessary for managing challenges and achieving goals in the university context (Robbins et al., 2004). Their role becomes even more significant when considering their impact on students with SLD (Livingston et al., 2018).

The primary aim of this study is to explore the emotional and motivational aspects of university students with SLD. While research and clinical practice have extensively examined the cognitive aspects of SLD, there is a significant need to investigate the emotional and motivational dimensions. This study employs a comprehensive set of self-report psychological questionnaires to assess anxiety, depression, resilience, academic motivation, cognitive strategies, and perfectionism. By doing so, this research provides a broader perspective compared to previous studies that mainly focused on cognitive aspects and their effect on academic performance or on isolated emotional and motivational factors. This multidimensional approach highlights the diverse characteristics of university students with SLD.

Moreover, while many studies have concentrated on children and adolescents with SLD, there is a relative paucity of research on university students, who are increasingly represented in Italian universities. A more in-depth understanding of the emotional and motivational profiles of university students with SLD might reveal potential areas for early intervention and prevention. This could have significant implications for national education and health systems, particularly within the Italian educational framework. Identifying and addressing emotional and motivational challenges early on can mitigate the risk of academic setbacks and promote a positive learning experience.

## 2 Method

### 2.1 Participants

We assessed 61 university students, 32 with SLD and 29 in the control group. The SLD group consisted of 32 students (13 males and 19 females; mean age = 23.6 years, SD = 3.3) who had been diagnosed with a SLD according to the DSM-V criteria (American Psychiatric Association, 2013) and adhering to the typically adopted guidelines for psychodiagnostic assessment (see Guidelines on the management of Specific Learning Disorders, 2022), which include: a normal level of general intelligence, clinically significant performance difficulties in reading and/or writing and/or mathematics and no neurological or sensory deficits that could account for their difficulties. No student had disability certificates or other psychopathological disorders. Students with comorbid ADHD were excluded.

The SLD group included the following diagnoses: dyslexia (*N* = 9), dysgraphia (*N* = 1), dyscalculia (*N* = 3), comorbidity of dyslexia and dysgraphia (*N* = 8), comorbidity of dyslexia and dyscalculia (*N* = 3), and comorbidity of dyslexia, dysgraphia, and dyscalculia (*N* = 8).

These students voluntarily approached the specialized university center, which provided a specific program for students with learning disorders. Of the total, 10 students with an early diagnosis were reassessed and had their diagnoses confirmed. The other 22 students, who recognized characteristics associated with a SLD, were regularly assessed by the university center and received diagnoses. Among the group of students with SLD, 62.5% attended a graduate program in the humanities and social sciences area, while 34.4% attended a graduate program in the scientific and technological area.

The SLD students were compared with a control group of 29 typically developing university students. (8 males and 21 females; mean age = 23.00, SD = 2.3). Among the control group, 86.2% attended a graduate program in the humanities and social sciences area, 10.3% attended a graduate program in the scientific and technological area, and 3.5% attended a graduate program in the healthcare area.

All the participants attended different years of study. Table 1 shows the results of the administered tasks, to both groups, to measure their cognitive profile (WAIS-IV; Wechsler, 2013) and reading, writing, and arithmetic abilities (LSC-SUA; Montesano et al., 2020). The groups were matched for chronological age [*F*(1,59) = 0.94; *p* 0.34] and General Ability Index- GAI [*F*(1,59) = 3.21; *p* 0.08], as measured by the Wechsler Intelligence Scale for Children, Fourth Edition (WAIS-IV; Wechsler, 2013). Considering these patients, the GAI might be deemed a more suitable measure of intelligence compared to the Full-Scale Intelligence Quotient (FSIQ). The FSIQ could be adversely influenced by a decline in both the Working Memory Index and the Processing Speed Index, typically impaired in adults with SLD and other neurodevelopmental conditions (e.g., ADHD) (Theiling and Petermann, 2016; Scorza et al., 2023).

**Table 1:** Differences between students with SLD and those typical development (control group) in age, cognitive profile and reading, writing, and calculation tasks.

	**SLD group M(SD)**	**Control group M(SD)**	***F* (1,59)**	***p***	**η**^**2**^**p**
Age	23.8 (0.58)	23 (0.61)	0.94	n.s	0.02
FISQ (WAIS-IV)	100 (2.32)	113 (2.22)	16.2	<0.001	0.22
GAI (WAIS-IV)	105 (2.28)	111 (2.18)	3.21	n.s.	0.05
Vinegrad plus	13.72 (0.69)	3.45 (0.73)	104	<0.001	0.64
Reading text syllable/s.	4.41 (0.12)	5.92 (0.12)	85.3	<0.001	0.59
Reading text errors	6.61 (0.57)	2.16 (0.55)	34.3	<0.001	0.37
Reading words syllable/s.	3.23 (0.13)	4.31 (0.12)	39.3	<0.001	0.40
Reading words errors	3.66 (0.43)	0.58 (0.41)	28.9	<0.001	0.33
Reading non-words syllable/s.	1.94 (0.09)	2.87 (0.08)	65.8	<0.001	0.53
Reading non-words errors	5.53 (0.68)	2.26 (0.65)	13.3	<0.001	0.18
Reading comprehension	10.1 (0.41)	10.7 (0.39)	1.21	n.s.	0.02
Dictation words errors	4.28 (0.52)	1.42 (0.50)	17.2	<0.001	0.23
Dictation words with articulator suppression errors	16.41 (1.22)	5.05 (1.17)	49.7	<0.001	0.46
Dictation text errors	6.84 (0.53)	3.00 (0.51)	29.7	<0.001	0.33
Math facts accuracy	14.2 (0.90)	26.2 (0.87)	101	<0.001	0.63
Mental calculation sec.	225 (9.26)	154 (8.87)	33.6	<0.001	0.36
Mental calculation accuracy	3.03 (0.40)	6.63 (0.39)	45.2	<0.001	0.43
Dictation numbers errors	4.32 (0.53)	1.47 (0.50)	16.9	<0.001	0.23
Reading numbers sec.	69.6 (3.62)	48.4 (3.46)	19.8	<0.001	0.25
Reading numbers errors	2.12 (0.57)	0.32 (0.54)	5.80	<0.05	0.09

FSIQ, full scale intelligence quotient; GAI, general ability index.

The study was approved by the Ethics Committee for Research in Psychology of the DiSUS and by the Data Protection Officer (DPO) of the University; the aims of the study were explained to the students, who provided written authorization for their participation in the research.

### 2.2 Procedure and material

All participants completed a battery of self-report psychological questionnaires in the presence of the experimenter. The time required for filling out the questionnaires was approximately 60 min.

#### 2.2.1 State–trait anxiety inventory (STAI-Y)

The STAI-Y (Spielberger, 1989) is an easily administered and interpreted tool for assessing and measuring anxiety. The questionnaire consists of 40 items, to which the individual responds in terms of intensity (ranging from “almost never” to “almost always”). The items are grouped into two scales focusing on how individuals generally feel or what they experience in specific moments. These two scales are:

- State anxiety (STAI-Y form 1): this scale conceptualizes anxiety as a specific experience, a feeling of insecurity, helplessness in the face of perceived harm that can lead to either worry or escape and avoidance.
- Trait anxiety (STAI-Y form 2): this scale represents a tendency to perceive stressful situations as dangerous and threatening, responding to various situations with varying intensity.

#### 2.2.2 Beck depression inventory-II

The Beck Depression Inventory-II (BDI-II; Beck et al., 1996); Italian version by Ghisi et al. (2006) is a self-report tool developed as an indicator of the presence and intensity of depressive symptoms. The test, consisting of 21 items, yields a total score and two scores related to the following areas:

- Somatic-Affective: pertaining to the somatic-affective manifestations of depression (loss of interest, loss of energy, changes in sleep and appetite, etc.);
- Cognitive: relating to cognitive manifestations (pessimism, feelings of guilt, self-criticism, etc.).

#### 2.2.3 Anxiety and resilience questionnaire

The Anxiety and Resilience Questionnaire (QAR) is part of the AMOS battery – Study Skills and Motivation: Assessment and Guidance Tests for Upper Secondary School and University (De Beni et al., 2014). The QAR is a comprehensive tool designed to assess the level of anxiety in study situations (anxiety factor) and, at the same time, the student’s ability to tackle particularly challenging study situations (resilience factor). It consists of 14 items, and response choices for each item are rated on a 5-point scale ranging from 1 (not at all) to 5 (completely).

#### 2.2.4 Academic motivation scale

The Academic Motivation Scale (AMS; Alivernini and Lucidi, 2008), developed in relation to the self-determination theory (Vallerand et al., 1992, 1993), consists of the following five subscales centered on the reason for choosing a degree program:

- Lack of motivation (or a-motivation): sample responses include: “Honestly, I do not know.”; “I feel like I’m wasting my time at school.”;
- External motivation: “To have better job prospects later on.”;
- Introjected motivation: “Because when I succeed at school, I feel important.”;
- Identified motivation: “Because I believe that a higher education will better prepare me for the career I have chosen.”;
- Intrinsic motivation: “Because I experience pleasure and satisfaction from learning new things...”

Response choices for each item are rated on an 11-point scale ranging from 0 (not true at all) to 10 (completely true).

#### 2.2.5 Self-regulated knowledge (cognitive/study strategies)

The Self-Regulated Knowledge Scale – University (SRKS-U; Italian translation “Scala di Auto-Regolazione degli Apprendimenti – Università” or SARA-U) was developed based on Pintrich’s self-regulated learning theory (Pintrich, 2004) and validated in Italy by Manganelli et al. (2015). The SARA-U consists of five subscales, each composed of three items that answer the question “When you study, how often do you do the following things?.” These five subscales assess the use of the following cognitive processes:

- Knowledge extraction: frequency with which students select information they consider most important;
- Knowledge linking: frequency with which students attempt to connect new knowledge with what they already possess;
- Knowledge training: frequency with which students put their knowledge into practice;
- Knowledge critique: frequency with which students ask questions and critically evaluate what they have learned, forming their own opinions;
- Knowledge monitoring: frequency with which students monitor their own knowledge.

The scale is used to measure the frequency with which students employ various cognitive strategies on a 5-point scale (1 = Never, 2 = Rarely, 3 = Sometimes, 4 = Often, 5 = Almost always).

#### 2.2.6 Multidimensional perfectionism scale (MPS)

The Multidimensional Perfectionism Scale – Short Form (MPS-SF; Hewitt et al., 1991) explores three defined dimensions of perfectionism:

- Self-oriented perfectionism (SOP): a form of perfectionism directed towards oneself, where the individual demands perfection from themselves;
- Other-oriented perfectionism (OOP): perfectionism directed towards others, characterized by a tendency to expect perfection from them;
- Socially prescribed perfectionism (SPP): a form of perfectionism where the central belief is that others expect perfection only from the individual.

The MPS-SF is a questionnaire consisting of 15 items on a Likert scale (from 1 to 7) that assess the level of agreement or disagreement with the statements provided. Higher scores indicate higher levels of trait perfectionism.

### 2.3 Data analysis

Data analyses were conducted with Jamovi Version 2.3.18.0 software. The Analysis of Covariance (ANCOVA) and Multivariate Analysis of Covariance (MANCOVA) were performed to compare the self-assessment questionnaire scores between the groups. Separate analyses were conducted on the scores obtained from individual self-assessment questionnaires included as dependent variables, while the group variable was used as an independent variable (fixed factor). Additionally, to control for gender effects, this factor was added as a covariate.

## 3 Results

One-way ANCOVA, controlling for gender effect, reveals a significant difference between the two groups (SLD vs. Control) for the *State–Trait Anxiety Inventory* Form 2 (STAI-Y 2) [*F*_(1,58)_ = 8.31; *p* < 0.05], with higher scores observed in university students with SLD compared to the control group (SLD M: 49.9, SD: 1.85; Control group M: 42.1, SD: 2.02). Although not reaching statistical significant difference between the SLD group and the control group (SLD M: 46.8, SD: 2.06; Control group M: 41.0, SD: 2.25), the scores on STAI-Y Form 1 tend to approach significance [*F*_(1,58)_ = 3.78; *p* = 0.057].

**Figure 1:**
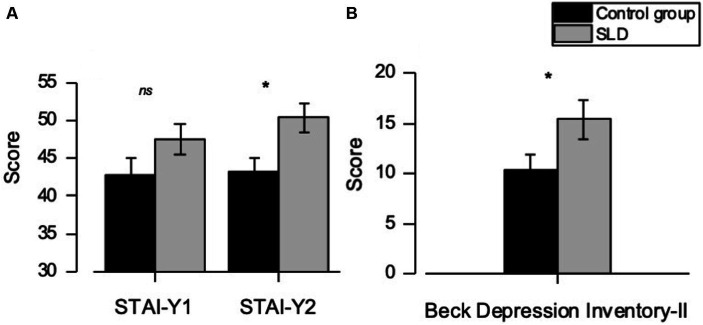
Scores obtained in the: **(A)** STAI-Y Form 1 and 2 and **(B)** BDI-II questionnaires in both examined groups. Error bars correspond to ±1 S.E.M. Stars refer to significance of *p*-values: ***p < 0.001, **p < 0.01, *p < 0.05 and ns, non-significant.

The main effect of group was also found to be significant for the *Beck Depression Inventory-II* (BDI-II) [*F*_(1,58)_ = 5.93; *p* < 0.05], with higher scores for the SLD group compared to the control group. (SLD M: 14.78, SD: 1.66; Control group M: 8.92, SD: 1.82). In the SLD group, 46.9% exhibited a clinical score exceeding the cut-off (cut-off = 15) on the BDI-II; in the control group, 27.6% scored above the cut-off. The results obtained from the two clinical scales (STAI-Y 2 and BDI-II) are shown in Figure 1.

**Figure 2:**
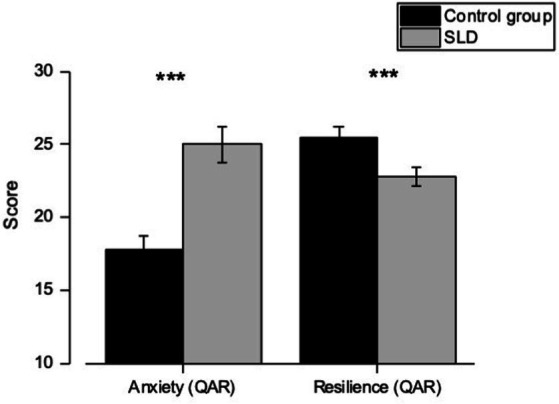
Scores obtained in the Anxiety and Resilience Questionnaire (QAR) for both examined groups. Error bars correspond to ±1 S.E.M. Stars indicate the significance of *p*-values: ***p < 0.001, **p < 0.01, *p < 0.05, and ns, for non-significant.

For the *Anxiety and Resilience Questionnaire* (QAR), a statistically significant difference emerges for both the anxiety factor [*F*_(1,58)_ = 24.02; *p* < 0.001] with higher scores for the SLD group compared to the control group, and the resilience factor [*F*_(1,58)_ = 7.53; *p* < 0.05] where the SLD group reports lower scores compared to the control group (Figure 2).

As shown in Table 2, the one-way multivariate analysis of covariance (MANCOVA), controlling for gender effects, reveals a statistically significant difference in the Academic Motivation Scale (AMS). Specifically, the difference is observed only for the subscale “intrinsic motivation” [*F*(1,58) = 4.38; *p* < 0.05], indicating lower scores for the SLD group compared to the control group. At the Self-regulated knowledge (SARA-U), the group effect was found to be significant for the cognitive strategies “knowledge linking” [*F*(1,63) = 7.05; *p* < 0.05], “knowledge training” [*F*(1,63) = 7.14; *p* < 0.05] and “knowledge criticism” [*F*(1,63) = 5.38; *p* < 0.05] with lower scores for the SLD group in all three strategies compared to the control group. No statistically significant differences emerge for any of the investigated dimensions in the Multidimensional Perfectionism Scale (MPS).

**Table 2:** Differences between students with SLD and those with typical development in motivational questionnaires (AMS, SARA-U, and MPS).

	**SLD group M(SD)**	**Control group M(SD)**	***F* (1,58)**	**p**
**Academic motivation scale (AMS)**				
Lack of motivation	3.16 (0.69)	2.00 (0.76)	1.26	n.s.
External motivation	5.44 (1.40)	3.55 (1.53)	0.66	n.s
Introjected motivation	21.2 (1.70)	18.6 (1.86)	0.94	n.s.
Identified motivation	32.8 (1.46)	34.9 (1.59)	1.42	n.s
Intrinsic motivation	31.7 (1.23)	35.1 (1.34)	4.38	<0.05
**Self-regulated knowledge (SARA-U)**				
Knowledge extraction	11.3 (0.38)	11.6 (0.42)	0.23	n.s.
Knowledge linking	11.4 (0.31)	12.6 (0.34)	6.59	<0.05
Knowledge training	10.7 (0.35)	11.9 (0.38)	6.41	<0.05
Knowledge critique	11.0 (0.35)	12.1 (0.38)	5.11	<0.05
Knowledge monitoring	10.5 (0.34)	11.3 (0.37)	2.75	n.s.
**Multidimensional perfectionism scale (MPS)**				
Self-oriented perfectionism	23.9 (1.13)	22.0 (1.23)	1.22	n.s
Other-oriented perfectionism	18.7 (1.13)	17.8 (1.24)	0.27	n.s
Socially prescribed perfectionism	18.2 (1.15)	15.9 (1.26)	1.29	n.s

## 4 Discussion

The main aim of this research was to examine the emotional and motivational aspects of university students with SLD and determine if they experience more significant psychological challenges than the control group. The findings highlighted that young adults with SLD demonstrate higher levels of anxiety compared to the control group. Specifically, these students display high trait anxiety, which is a stable and enduring personality trait that characterizes the individual consistently, regardless of a specific situation, as well as anxiety related to study situations. These findings are consistent with other studies in the literature that indicate that both in adulthood (Carrol and Iles, 2006; Davis et al., 2009; Nelson and Harwood, 2011; Bonuomo et al., 2023) and during childhood (Svetaz et al., 2000; Dahle et al., 2011), students with SLD experience internalizing problems.

Furthermore, the findings emphasize that young adults with Specific Learning Disabilities (SLD) experience higher levels of depression compared to the control group. These results are consistent with other studies in the literature that have noted a noteworthy rise in depressive symptoms among students with SLD when compared to their typically developing peers (Goldston et al., 2007; Wilson et al., 2009; Dahle et al., 2011; Re et al., 2014).

Furthermore, in addition to the increase in internalizing symptoms, our SLD group demonstrated diminished resilience scores, indicating a lower capacity among students to cope with particularly challenging situations when compared to the control group. These data align with other reports of young adults with dyslexia exhibiting lower levels of resilience (Scanlon and Mellard, 2002). Additionally, the SLD group had a higher number of students with late diagnosis who may exhibit fewer positive coping or adaptation strategies. They also did not have access to various protective factors, such as enhancement treatments aimed at improving their reading and writing skills, as well as adequate social support from family and school, that could have facilitated their ability to tackle particularly challenging study situations. Studies in the literature indeed show that in students with learning difficulties, family support and social factors, such as seeking help, can better predict university persistence and success (Trainin and Swanson, 2005; DaDeppo, 2009; Nalavany et al., 2011; Showers and Kinsman, 2017). This overall data demonstrates how resilience can potentially serve as a protective factor, as also evidenced in the literature (Stack-Cutler et al., 2014; Ghisi et al., 2016).

Several studies have investigated the role of motivation and cognitive strategies in positively influencing students’ academic performance (Diseth and Kobbeltvedt, 2010; Heikkila et al., 2011; Richardson et al., 2012) and in preventing the phenomenon of university drop-out (De Marco and Albanese, 2009; Biasi et al., 2017). The investigation into the role of motivation and cognitive strategies in academic performance and dropout prevention is a valuable addition, providing practical implications for educational settings. Group differences emerge in the intrinsic motivation subscale of the AMS, indicating when students engage in a learning activity for its own sake, purely for the pleasure of learning. The SLD group shows lower scores, indicating less engagement in a study activity driven by cognitive curiosity. Conversely, extrinsic motivation entails a higher risk of drop-out: elevated levels of intrinsic motivation lead to a reduction in this risk. Additionally, intrinsic motivation is positively associated with deep learning processes and the use of specific cognitive strategies (Vansteenkiste et al., 2009; Baeten et al., 2013; Biasi et al., 2017).

Concerning cognitive strategies, our findings indicate significantly lower scores in the SLD group for knowledge linking, knowledge training, and knowledge critical. This suggests that students with SLD engage less frequently in activities like connecting new knowledge with existing knowledge, applying their knowledge, asking questions, and critically assessing what they have learned to form their own understanding - crucial aspects for academic success. Biasi et al. (2017) demonstrate that university students who achieve high exam scores have higher scores in these cognitive strategies. This essentially indicates, as expected, a better study methodology employed by students who manage to achieve good results. University students with or without SLD do not exhibit statistically significant differences in terms of perfectionism. What emerges from a qualitative analysis is that both groups show higher scores for the self-oriented perfectionism dimension of the MPS-SF, highlighting a form of perfectionism directed towards oneself. In an academic context, this is associated with greater productivity, career success, and conscientiousness. The key to success in academic endeavors for students with a SLD appears to depend more on motivational factors than cognitive ones (Lockiewicz et al., 2014). For students with SLD who choose to pursue higher education, an additional personal commitment and appropriate strategies are required to overcome the lingering difficulties, despite compensatory functional development processes, affecting psychological well-being. Our results show and confirm that psychological consequences (anxiety, low resilience, etc.) and motivational issues are present in university students with SLD and can impact their well-being and quality of life. It is important to note that many students with SLD arrive at university without having received a diagnosis, which can influence academic and/or career choices, contribute to frequent dropouts, and lead to secondary problems in the psychopathological sphere.

In conclusion, it remains critically important to consider early learning screenings to prevent the psychological effects associated with late diagnosis (Iaia, 2018). Given the challenges that university students with SLD continue to face in completing their studies (Ghisi et al., 2016), targeted support interventions appear to be particularly relevant in terms of reducing perceived anxiety levels, increasing resilience, and improving academic performance (Derba et al., 2019). In light of these findings, implementing university services focused on preventing and managing psychological difficulties in students with SLD is strongly recommended.

## Data availability statement

The raw data supporting the conclusions of this article will be made available by the authors, without undue reservation.

## Ethics statement

The studies involving humans were approved by Ethics Committee for Research in Psychology of the DiSUS and by the Data Protection Officer (DPO) of the University. The studies were conducted in accordance with the local legislation and institutional requirements. The participants provided their written informed consent to participate in this study.

## Author contributions

MI: Conceptualization, Data curation, Formal analysis, Methodology, Resources, Writing – original draft. FV: Investigation, Resources, Writing – review & editing. MC: Investigation, Resources, Writing – review & editing. MT: Formal analysis, Methodology, Writing – review & editing, Funding acquisition. CVM: Writing – review & editing, Funding acquisition. PA: Funding acquisition, Project administration, Supervision, Writing – review & editing.
